# Are probiotics effective in reducing the metabolic side effects of psychiatric medication? A scoping review of evidence from clinical studies

**DOI:** 10.1038/s41398-024-02735-z

**Published:** 2024-01-15

**Authors:** Sonja Mötteli, Stefan Vetter, Michael Colla, Florian Hotzy

**Affiliations:** Department of Psychiatry, Psychotherapy and Psychosomatics, Psychiatric Hospital of the University of Zurich, Zurich, Switzerland

**Keywords:** Schizophrenia, Clinical pharmacology

## Abstract

The psychopharmacological treatment of patients with schizophrenia or depression is often accompanied by serious side effects. In particular, the clinical findings of weight gain are worrying, as this side effect can lead to various medical sequelae in the future. However, the treatment of metabolic changes in psychiatric patients is often neglected or unsuccessful. An improved knowledge of possible therapeutic approaches is needed. The aim of this study was to provide an overview of the utilisation and effectiveness of probiotics in reducing weight gain in patients with severe mental illness. A scoping review of studies published until 15 June 2022 was conducted to identify studies using probiotics in people with schizophrenia or depression. We systematically searched the databases EMBASE, PubMed (MEDLINE), Web of Science and SCOPUS with a predefined search string. In addition, reference lists of relevant publications were examined for additional studies. The studies were assessed by two reviewers. The primary outcomes were weight-related measurements. The secondary outcomes were metabolic blood parameters and gut microbiota. Four studies ultimately met the inclusion criteria. Two studies in which probiotics were administered did not find significant effects on pharmacologically induced weight gain. The other two studies examined the effects of synbiotics (a combination of probiotics and prebiotics). Interestingly, less weight gain was observed in individuals with this combined intervention. Adjustments in diet can be helpful and are generally well-accepted interventions in the fight against pharmacologically induced weight gain. The clinical use of probiotics and prebiotics (or synbiotics) as dietary interventions may represent a promising additional strategy in this regard. However, the few studies available showed no clear conclusions.

## Introduction

Individuals with severe mental illness (SMI), such as chronic depression and schizophrenia, have an increased risk of obesity and metabolic complications [1, 2]. This results in a decreased quality of life and reduced life expectancy of up to 25 years [3, 4]. Several factors contribute to weight-related problems in this population.

In addition to unhealthy eating habits and reduced physical activity [4–6], it is mainly the substance groups of antipsychotics and antidepressants that are known to cause side effects, such as increased appetite, weight gain and long-term metabolic syndrome [7–10]. An unhealthy eating style, high blood LDL cholesterol, high body mass index (BMI), or obesity (BMI (≥30 kg/m^2^), and high blood sugar are among the most frequent modifiable risk factors for cardiovascular diseases (CVD), which are a major cause for mortality and disability [11]. Besides that, obesity increases the risk for cancer, diabetes or fatty liver disease [12]. Therefore, metabolic side effects are a major concern regarding treatment with psychiatric medication. They are also a common reason for psychiatric patients to stop taking their medication [13]. This might reduce the risk for a metabolic syndrome, but it comes at the cost of an increased risk of a relapse, an exacerbation and a chronic course of the psychiatric disorder. This, in turn, is associated with a reduced quality of life and life expectancy.

In addition to the direct induction of appetite via neurocircuits [8], antipsychotics and antidepressants reduce microbial diversity [14, 15]. Reduced gut diversity is associated with weight gain and cardiovascular-related side effects [15, 16]. Such side effects might be harder to regulate through a reduction in caloric intake or an increase in physical activity, because the altered gut bacterial composition might cause an increased energy harvest and storage [16].

In addition, some patterns of microbiotal composition seem to be associated with multiple diseases, including mental disorders [17, 18]. For instance, the microbiomes in patients with mental disorders, such as schizophrenia, bipolar disorders, depression, anxiety disorders, PTSD and eating disorders, were shown to have decreased diversity compared to the microbiome in healthy controls [19–23]. In general, patients with mental disorders were shown to have fewer bacterial genera that produce short-chain fatty acids (e.g. butyrate) and higher levels of lactic acid-producing bacteria, and bacteria associated with glutamate and GABA metabolism [24]. On a genus level, in patients with schizophrenia, *Prevotella* levels were higher and *Bacteroides*, *Haemophilus*, and *Streptococcus* were lower. In bipolar disorders, *Bifidobacterium* and *Oscillibacter* were higher. Patients with depression had higher levels of *Alistipes* and *Parabacteroides* and lower *Prevotella* [24], and the severity of depression was negatively correlated with *Clostridia* and *Firmicutes* [25].

Some, but not all, antipsychotics and antidepressants have been shown to have antibiotic effects [15, 26, 27]. This might be one reason that treatment with psychiatric medication is associated with a less diverse microbiome composition [14, 15], which, again, is associated with weight gain [28, 29]. Furthermore, the proportion of some bacteria stems in the microbiome correlated with the extent of antipsychotic-induced weight gain [15]. In addition, in patients with antipsychotic treatment, a decrease in specific bacterial species was associated with an increase in insulin resistance [30].

This prompted a discussion about whether probiotics might be effective in mitigating antipsychotic-induced side effects, such as weight gain, reduced insulin sensitivity and other metabolic parameters [31, 32]. Probiotics have been shown to normalise metabolic parameters in obese patients [33] and animal models of obesity [34, 35]. This indicates that probiotic-induced changes in microbiota composition might be beneficial for body weight. Despite the potential benefits of probiotics regarding antipsychotic-induced side effects, studies on this topic with psychiatric patients are scarce. To evaluate the clinical relevance of probiotics for psychiatric treatment, a more profound knowledge of the present evidence regarding the interaction between probiotics, psychiatric medication and its side effects is necessary. Therefore, the aim of this study was to systematically identify and describe the studies that examined the effects of probiotics on antipsychotic-induced sight effects.

## Methods

A scoping review was conducted to provide a general overview of the few heterogeneous studies using probiotics in psychiatric patients to treat drug-induced side effects, such as weight gain, and to identify promising research directions in the complex field of current microbiome research.

Originally, a systematic review was planned, and the protocol was registered on the PROSPERO database (identifier CRD42022339340). Further, this study was conducted according to the Preferred Reporting Items for Systematic Reviews and Meta-analysis (PRISMA) statement [36]. However, due to the very limited availability of studies and the complexity of the topic, a scoping review was evaluated as a more appropriate first step to summarize existing research findings, to identify research gaps, and to derive recommendations for future studies in the field [37, 38]. We did not require ethical approval, as this review only involved secondary analyses of published data.

### Inclusion and exclusion criteria

The Population, Intervention, Comparison, Outcomes, and Study (PICOS) framework [39] was applied to define the study and eligibility criteria:

- Population (P): Patients with mental disorders such as depression or schizophrenia, animal models
- Intervention or exposure (I): Probiotic or synbiotic
- Comparison (C): Placebo
- Outcome (O): Weight, metabolic parameters (BMI, blood), gut microbiome, mental health
- Study (S): randomized controlled trials
- Time frame (T): database inception–2022

We restricted the search to studies published in English and German and in peer-reviewed journals. We included original research papers only from database inception until 15 June 2022. Case reports or series, (systematic or narrative) reviews, expert opinion papers, conference abstracts and editorials were excluded. Because the literature on the study’s topic was scarce, we applied a strategy in which research on humans as well as on animals could be included if the inclusion criteria were met.

### Search strategy

We conducted a search in the following databases: EMBASE, PubMed (MEDLINE), Web of Science and SCOPUS. In a preliminary unstructured database screening, we found that the literature on our study question was scarce. Therefore, we kept the search strategy open and applied no restrictions to the process. For example, one database also presented conference abstracts or review articles. The search was conducted on 15 June 2022.

The search terms were developed by the authors and a search string was built in consultation with a specialised librarian of the medical department of the University of Zurich. Where possible, we used MESH terms (EMBASE, PubMed (MEDLINE)) besides title and abstract screening.

We searched the databases for publications on side effects regarding weight and metabolic parameters, psychiatric medication, probiotics or synbiotics and mental disorders. The specific search strings for the different databases were registered in PROSPERO and are outlined in the supplementary material. In addition to database screening, we reviewed the bibliographies of the included studies for additional relevant studies [40].

### Study selection and quality assessment

**Fig. 1:**
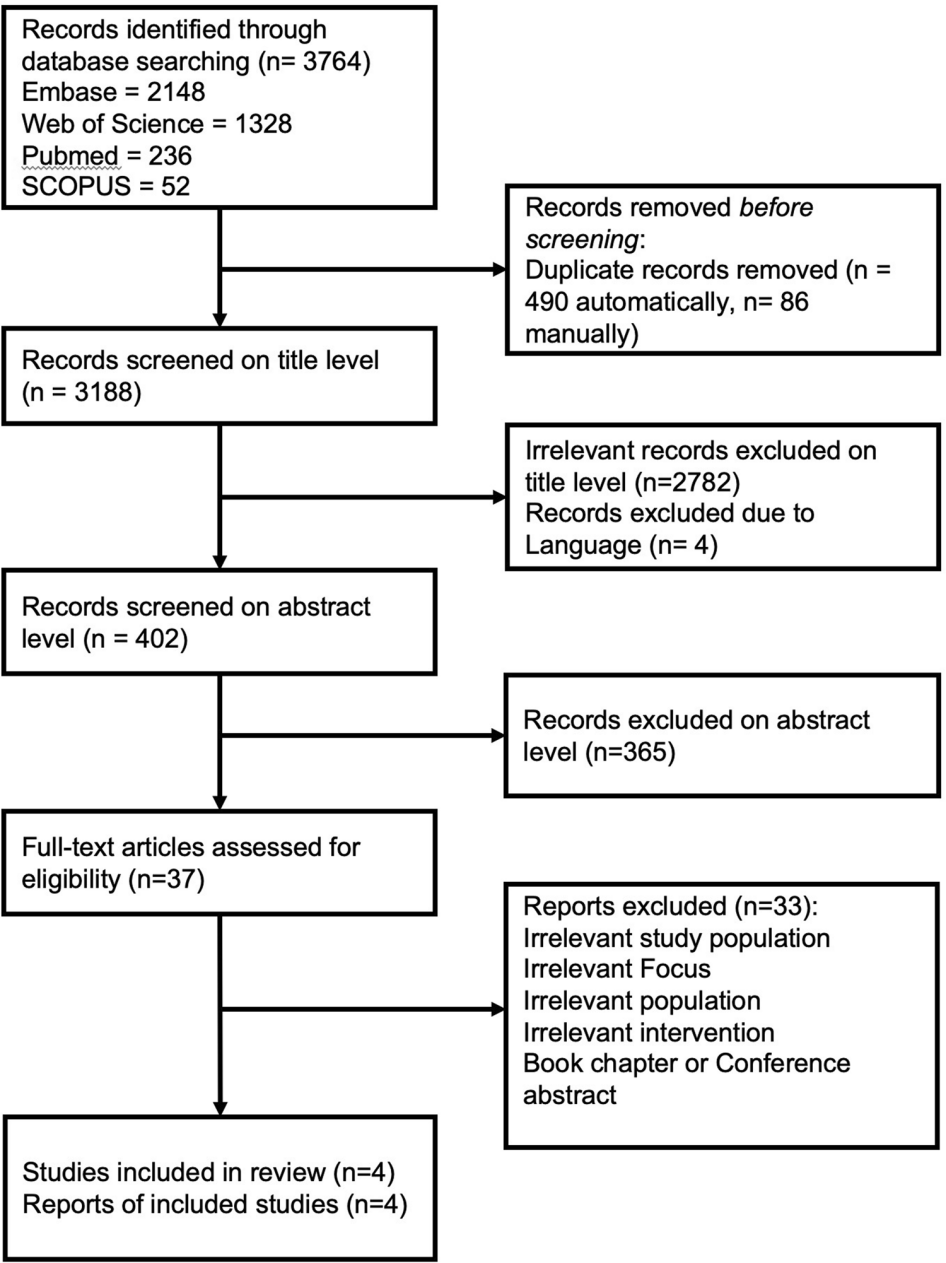
PRISMA flow diagram for the search strategy.

We downloaded all search results and imported them into a reference management system (Endnote v. X9). After merging all findings (Fig. 1), we deleted duplicates (*n* = 576) and non-English publications (*n* = 4). The remaining publications were independently screened for eligibility by one author (FH) and one research assistant (TD). Any disagreements regarding the in- or exclusion of publications were discussed with another author (SM) and together a consensual decision was made in the extended team. The quality of the studies was independently assessed by FH and TD, using a modified version of the randomised trial quality checklist as suggested by Wright et al. [40] and the critical appraisal skills programme (CASP) randomised controlled trial checklist [41]. Risk of Bias was assessed using the Revised Cochrane risk-of-bias tool for randomized trials (RoB 2) [42].

### Data extraction

Following the recommendations for systematic reviews [40, 41], we extracted the following characteristics from the included studies: study design, study aims, definition of in- or exclusion criteria, recruitment procedure, information on dropouts and final sample size, study intervention, bacterial composition of the applied probiotic, outcome measures, study length, study results regarding weight, (if available, laboratory parameters, psychiatric symptoms and gut microbiome) and funding statement. The studies’ results were summarised in a narrative synthesis.

## Results

The search procedure yielded 3764 publications. After the title and abstract screening, full-text articles were accessed for 37 records. Of these, 33 were subsequently excluded and four met the inclusion criteria. No animal study met our inclusion criteria (for details, see Fig. 1, PRISMA flow diagram for the search strategy).

### Quality and characteristics of the included studies

All (*n* = 4) studies were randomised trials conducted in humans. Two of them were double blind. All publications had clearly defined aims. In all studies, the inclusion or exclusion criteria and recruitment procedures were described appropriately. Three (*n* = 3) of the studies used a sample size calculation or justified the number of participants [43–45].

One study included psychiatric inpatients [46], one study included psychiatric outpatients [43]; for two studies, the recruitment setting was not specified [44, 45].

The sample sizes ranged from 51 to 118 participants. The number of non-participations or dropouts was declared in all studies and ranged from two [44] to 18 [45].

The mean age of the participants was 23.9–45.2 years. All studies included patients with schizophrenia. Two studies also included patients with bipolar disorders [45] and schizoaffective disorders [46], respectively.

The nature of the funding sources was disclosed in all publications. More details on study characteristics and quality criteria are shown in Table 1.

**Table 1:** Characteristics and Quality criteria of the included studies.

Publication	Design	Run in/wash out periods	Aims	Inclusion		Dropouts	Final sample size (N)	Model fidelity	Intention-to-treat analysis	Study length (weeks)	Funding	Risk of bias^a^
				Diagnoses	Age (Years)							
Jamilian et al. [43]	Randomized, double-blind, placebo-controlled trial	N.I.	Evaluation of the effects of selenium and probiotic co-supplementation on clinical signs and metabolic status in patients with chronic schizophrenia.	Chronic schizophrenia	18–60	9	*n* = 26 selenium plus probiotics *n* = 25 placebo	Y	N.I.	12	Y	++
Huang et al. [44]	Randomized clinical trial	N.I.	Examination of whether probiotics or dietary fibre supplementation is useful for the metabolism in patients with schizophrenia, specifically regarding olanzapine-induced weight gain.	First episode schizophrenia	18–50	Study 1: 14 Study 2: 2	Study 1: *n* = 37 olanzapine *n* = 39 olanzapine plus probiotics Study 2: *n* = 28 olanzapine *n* = 30 olanzapine plus probiotics and dietary fibre	N.I.	N.I.	12	Y	+/−
Huang et al. [45]	Randomized, placebo-controlled, double-blind clinical trial	N.I.	Examination of whether probiotics or dietary fibre consumption added to antipsychotic treatment reduces weight gain and other metabolic side effects in patients with severe mental disorders.	Schizophrenia or bipolar disorder	18–45	18	*n* = 32 probiotics plus dietary fibre *n* = 29 probiotics *n* = 29 dietary fibre *n* = 28 placebo	Y	Y	12	Y	++
Yang et al. [46]	Randomized controlled study	N.I.	Examination whether can prevent olanzapine-induced weight gain and increased appetite.	First-episode schizophrenia and schizoaffective disorder	18–55	Y (n = 3)	*n* = 34 olanzapine monotherapy *n* = 33 olanzapine plus bifidobacterium	N.I.	N.I.	12	Y	+/−

^a^According to the Revised Cochrane risk-of-bias tool for randomized trials (RoB 2); *Y* Yes, *N* No, *N.I.* No information.

### Methods and results of the included studies

The interventions and statistical methods were described conclusively in all publications (Table 2). In each study, a probiotic with a specific bacterial composition was applied. On the genus level, *Bifidobacterium* (*n* = 4 studies), *Enterococcus* (*n* = 3 studies) and *Lactobacillus* (*n* = 4 studies) were administered with different species and combinations. In two studies, synbiotics (a combination of prebiotics and probiotics) were applied [44, 45]. All the studies made statements on adverse effects [43–46]. The application of probiotics and prebiotics was well tolerated; no study has reported adverse effects induced by probiotics.

**Table 2:** Methods and results of the included studies.

Publication	Outcome measures	Intervention		Study length	Results			
					Weight, Diet	Laboratory parameters	Psychiatric symptoms	Microbiome
Jamilian et al. [43]	Weight: Changes in weight/BMI, 3-day dietary records (nutritionist IV software) Psychopathology: PANSS, BPRS Blood: Fasting blood samples	Probiotic plus 200 μg/day selenium versus placebo	Probiotic: LactoCare contained Lactobacillus acidophilus, Bifidobacterium lactis, Bifidobacterium bifidum, and Bifidobacterium longum, each strain 2 × 10^9^ CFU/day Prebiotic: Selenium yeast 200 μg/day	12 weeks	No significant difference in weight gain in controls (+0.2 kg; SD = 0.8) and the probiotic and selenium group (+0.5 kg; SD = 1.8).	Significant elevation in TAC, GSH, QUICKI and reduction in CRP, FPG, insulin levels and HOMA-IR in patients with probiotic and selenium	Significant improvement in the PANSS score in patients with probiotic and selenium	n.a.
Huang et al. [44]	Weight: Changes in weight/BMI Blood: Insulin, insulin resistance index (IRI), fasting glucose, lipid metabolism	Study 1: 12-week olanzapine (15–20 mg/day) plus probiotics (840 mg twice daily) versus olanzapine monotherapy Study 2: 12-week olanzapine (15–20 mg/day) plus probiotics (840 mg twice daily) and dietary fibre (30 g twice daily) versus olanzapine monotherapy	Probiotic: Bifico containing live Bifidobacterium, Lactobacillus and Enterococcus at concentrations of ≥ 5.0 × 10^7^ CFU/day. Prebiotic: 10 g of bitter melon (Momordica charantia) and oligosaccharides (fructooligosaccharides and oligoisomaltoses), 20 g of kudzu starch, insulin and resistant dextrin	12 weeks	Probiotics plus dietary fibre significantly attenuated olanzapine-induced weight gain (+5.1 kg; SD = 3.5) compared to controls (+9.2 kg; SD = 3.8) while retaining its psychopathological effects; probiotics alone did not prevent weight gain.	Increases in fasting insulin and IRI were higher in the olanzapine monotherapy group in both studies	Psychopathological symptoms improved in all groups after 12 weeks	n.a.
Huang et al. [45]	Weight: Weight and height, Psychopathology: PANSS, HAMD, YMRS Blood: Fasting glucose, insulin, lipid profiles, liver and renal function Other: Gut microbiome, adverse events (TESS)	Four groups taking atypical antipsychotics: Probiotics (1680 mg/d) plus dietary fibre (60 g/d) Probiotics (1,680 mg/d plus dietary fibre placebo) Probiotic placebo plus dietary fibre (60 g/d) Probiotic placebo plus dietary fibre placebo	Probiotic: Bifico (1680 mg/d) containing live Bifidobacterium 1.7 × 10^9^ CFU/g, Lactobacillus 3.8 × 10^8^ CFU/g, and Enterococcus 7.8 × 10^8^ CFU/g Dietary fibre: 10 g hi-fibre drink plus 20 g extra herb powder	12 weeks	Probiotics plus dietary fibre significantly reduced weight (−2.4 kg; CI:1.3–3.4) while it increased in the placebo group (2.6 kg; CI:0–4.2). Probiotics or dietary fibre alone prevented further weight gain.	Probiotics plus dietary fibre significantly prevented further metabolic disturbances related to insulin, fasting glucose, and lipid profiles	n.a.	Combination of probiotics and dietary fibre was associated with a decreased abundance of Firmicutes and increased abundance of Bacteroidetes, Bacteroidaceae, Rikenellaceae, and Tannerellaceae
Yang et al. [46]	Weight: Changes in weight/BMI, appetite Psychopathology: Symptomatology incl. PANSS	Olanzapine with probiotics versus olanzapine monotherapy	Probiotic: Capsules of live Lactobacillus acidophilus, Bifidobacterium longum and Enterococcus faecalis each strain 3 × 10^9^ CFU/day	12 weeks	Controls (+7.9 kg; SD = 4.2) and olanzapine plus probiotics group (+7.4 kg; SD = 4.4) did not significantly differ in appetite and weight gain. Increased appetite levels were associated with greater weight gain.	n.a.	Similar improvements in the PANSS score in both groups	n.a.

*BMI* body mass index, *BPRS* Brief Psychiatric Rating Scale, *HAMD* Hamilton Depression Scale, *PANSS* Positive and Negative Syndrome Scale, *YMRS* Young Mania Rating Scale, *TESS* Treatment Emergent Symptom Scale, *CRP* C-reactive protein, *FPG* fasting plasma glucose, *GSH* glutathione, *HOMA-IR* homeostasis model of assessment-insulin resistance, *IRI* insulin resistance index, *QUICKI* insulin sensitivity check index, *TAC* total antioxidant capacity.

Two studies explicitly measured the effect of probiotics on olanzapine-induced weight gain [44, 46], one study also included patients with antiepileptic drugs [45], and one study did not describe the antipsychotics applied in detail [43].

The outcome measures were described conclusively in all publications. All studies assessed the influence of probiotics on body weight. One study measured the effect on the gut microbiome, three on laboratory parameters and three on psychiatric symptoms (Table 2).

Yang et al. [46] used a probiotic preparation that included stems of live *Lactobacillus acidophilus*, *Bifidobacterium longum* and *Enterococcus faecalis*. In this study, a smaller weight gain in the probiotic group during the first study weeks disappeared at weeks 8 and 12. Finally, probiotics had no significant effect anymore on weight gain compared to the placebo group.

In the study by Jamilian et al. [43], the probiotic preparation contained a combination of *Bifidobacterium bifidum*, *lactis* and *longum* and *Lactobacillus acidophilus*. This study found no significant weight change in the probiotic group compared to the placebo group. Nevertheless, the intake of probiotics was associated with better metabolic parameters and reduced CRP in the blood.

In the studies by Huang et al. [44, 45], a probiotic preparation (Bifico) that included stems of live *Lactobacillus acidophilus*, *Bifidobacterium longum*, and *Enterococcus faecalis* was applied. In both studies, the intake of the probiotic preparation alone had no significant effects on body weight. In contrast, the application of synbiotics (probiotic and dietary fibre) resulted in significant weight loss in both studies.

Psychopathology improved during the study period. In one study [43], the clinical improvement was higher in the intervention group with probiotics and selenium.

Only one study [45] analysed the gut microbiome and found that the application of a combination of probiotics and dietary fibre was associated with changes in microbiome composition. The details are shown in Table 2.

## Discussion

This scoping review identified four studies on the effects of probiotics on weight gain and other metabolic side effects in individuals with schizophrenia or bipolar disorders treated with psychiatric medication. In two studies that applied probiotics only, no significant effect on weight was found at the end of the studies [43, 46]. However, the intake of synbiotics resulted in a significant reduction of weight gain and improvement of other metabolic parameters, which is discussed to be driven by shifts in the gut microbiome towards bacteria stems with a more advantageous food metabolism profile [44, 45]. The augmentation with this combination during the intake of psychiatric medication might be a promising approach for further investigation.

Psychiatric medication (including antidepressants, antipsychotics or mood stabilisers) often induces metabolic side effects, such as weight gain [7–9]. Lifestyle interventions, such as the improvement of dietary habits in people with SMI, have shown limited evidence to improve physical health conditions [47]. Such interventions require sustained adherence and persistence, which is not always possible in psychiatric crises or chronic illnesses. Therefore, it is advisable to use different strategies simultaneously to improve the metabolic situation. On a pharmacological level, metformin has been shown to have positive effects on the weight of patients with antipsychotic medication [48]. Nevertheless, although its use is recommended early in antipsychotic-induced weight gain, it is not broadly used, has side effects, and it has to be prescribed “off-label” for antipsychotic-induced weight gain [49]. In addition, GLP-1 agonists, such as liraglutide are effective in decreasing weight, but some have to be injected regularly and they might have adverse events [50].

In contrast, probiotics are not only well tolerated [51] but also have high acceptance due to their natural origin. In addition, they might have positive effects on mental health problems, such as depression or anxiety [51, 52]. Another important and positive characteristic of probiotics is that they do not seem to impede the effects of psychiatric medication on the central nervous system [53]. Therefore, they are considered promising supplements in psychiatric treatment [54]. The results of this review showed that the intake of probiotics alone did not lead to a significant reduction in antipsychotic-induced weight gain [43, 46]. Nevertheless, two studies showed promising results for synbiotics [44, 45].

In a study of rats, the application of prebiotics based on galacto-oligosaccharides attenuated olanzapine-induced weight gain [53]. The gut microbiota ferments these prebiotics into short-chain fatty acids (SCFA), including acetate, butyrate and propionate, which are discussed to induce weight loss [55]. Furthermore, a prebiotic-based diet with fermented rice has led to a normalisation of the metabolism in high-fat diet (HFD)-fed mice [56]. A study on patients with bipolar disorders and schizophrenia showed that the intake of a prebiotic (resistant starch) was not associated with significant weight changes but led to changes in the gut microbiome [57]. These differing study results imply that the potential effect of probiotics or prebiotics alone or in combination is embedded in a complex process. Only one of the included studies also analysed the effects of the probiotic intervention on the gut microbiome and found significantly increased numbers of observed species in the probiotics plus dietary fibre group, while there were no changes in the other three groups [45]. The assumption was that pre- or probiotics induce a different composition of the gut microbiome and, therefore, lead to changes in the metabolism of food [45].

In addition, there has been evidence that some bacterial stems, such as *Coriobacteriales* or *Akkermansia*, might be protective against weight gain [58, 59]. Weight loss following a high fat diet was also found after the application of probiotics that contained *Bifidobacterium adolescentis*, *Lactobacillus mucosae* and *Weissella cibaria* [60]. In the general population, a combination of diet and probiotics led to weight loss [33]. However, with only a few studies available on this topic, further studies are needed to gain more knowledge about the way probiotic or prebiotic compositions affect the microbiome and how this is associated with weight changes and other metabolic risk factors. In addition, a personalised approach might be helpful as the microbiota composition and its alterations by medication might differ on an individual level. This would include the analysis of the microbiome prior to and during a probiotic intervention, in addition to measuring several control variables, such as dietary habits and other lifestyle factors.

### Limitations

Only four studies met our inclusion criteria, which did not allow us to draw definitive conclusions about the effects of probiotics on weight gain and other metabolic side effects of psychiatric medication. Although all four studies had a randomised controlled study design, they included only small sample sizes and differed in their methodological approaches. For instance, different groups of patients were included, such as individuals with schizophrenia, bipolar or schizoaffective disorders, which were not analysed separately. In addition, we found no study on patients with depression that met our inclusion criteria. Pharmacological treatment of depression can induce weight gain, metabolic syndrome [10] and changes in the microbiome [61]. Further studies are needed to examine the effect of probiotic interventions in patients with antidepressant-induced weight gain. In addition, two of the reviewed studies included patients with first-time schizophrenia who were drug naive or at least had a short history of medication intake [44, 46]. Patients with chronic disorders are more likely to be affected by the side effects of pharmacological treatment; thus, they should be the target study population of future studies.

Furthermore, the probiotics applied differed on a genus and species level. Due to the small number of included articles, a direct comparison between the probiotics used was not possible.

Reduced weight gain, or even weigth loss was seen in persons with higher abundance of stems, such as *Coriobacteriales*, *Akkermansia*, *Bifidobacteria*, *Lactobacillae* and *Weissella* [58–60]. The bacterial stems used in the included studies differed slightly among the reviewed studies. This might be a reason for the lack of effects in the probiotic groups.

Future studies should aim to compare the potential of different bacteria stems with and without additional prebiotics, including personalised approaches based on individual microbiome compositions prior to the intervention.

### Conclusion

In conclusion, synbiotics were shown to reduce antipsychotic-induced weight gain in two studies. The augmentation with probiotics alone did not influence weight gain significantly. The intake of pro-, pre- and synbiotics was well tolerated and one study showed changes in the gut microbiota in the group with synbiotics. Further, synbiotics, but also probiotics were associated with healthier laboratory parameters. Especially synbiotics might be an effective strategy to reduce antipsychotic-induced weight gain. Nevertheless, the review is based on the four included studies and did not allow us to draw general conclusions about the effect of pro- or synbiotics on antipsychotic-induced side effects but indicated a clear need for more research in this new field.

### Supplementary information

Supplementary material
