# Supplementary material for: Are probiotics effective in reducing the metabolic side effects of psychiatric medication? A scoping review of evidence from clinical studies
Source: Transl Psychiatry. 2024 Jan 15;14:26. doi: 10.1038/s41398-024-02735-z (PMC10789870; doi:10.1038/s41398-024-02735-z)
Supplement: Supplementary file 1 — Supplementary material [file 41398_2024_2735_MOESM1_ESM.docx]

**Supplementary material: Are probiotics effective in reducing the metabolic side effects of psychiatric medication? A scoping review of evidence from clinical studies**

**Detailed search-Terms for the included Databases**

**EMBASE**

('side effect':ti,ab,kw OR 'adverse event':ti,ab,kw OR 'metabolic syndrome':ti,ab,kw OR 'metabolism':ti,ab,kw OR 'weight change':ti,ab,kw OR 'weight gain':ti,ab,kw OR 'weight loss':ti,ab,kw OR 'diabetes':ti,ab,kw OR 'cholesterol':ti,ab,kw OR 'hyperlipidemia':ti,ab,kw OR 'dyslipidemia':ti,ab,kw OR 'hba1c':ti,ab,kw OR 'overnutr*':ti,ab,kw OR 'malnutr*':ti,ab,kw OR 'obes*':ti,ab,kw

OR

'side effect'/exp OR 'adverse event'/exp OR 'adverse drug reaction'/exp OR 'metabolic syndrome x'/exp OR 'metabolism'/exp OR 'disorders of carbohydrate metabolism'/exp OR 'disorders of lipid and lipoprotein metabolism'/exp OR 'body weight change'/exp OR 'diabetes mellitus'/exp OR 'hyperlipidemia'/exp OR 'dyslipidemia'/exp OR 'glycosylated hemoglobin'/exp OR 'overnutrition'/exp OR 'malnutrition'/exp)

AND

('pharmacolog*':ti,ab,kw OR 'pharmaceutic*':ti,ab,kw OR 'medication':ti,ab,kw OR 'medication adherence':ti,ab,kw OR (psychopharm*:ti,ab,kw OR antidepress*:ti,ab,kw) AND neuroleptic:ti,ab,kw OR antipsychotic:ti,ab,kw

OR

'pharmacology'/exp OR 'drug therapy'/exp OR 'medication compliance'/exp OR 'drug therapy'/exp OR 'psychopharmacology'/exp OR 'antidepressant agent'/exp OR 'neuroleptic agent'/exp)

AND

('probiotic':ti,ab,kw OR 'synbiotic':ti,ab,kw OR Microbiome:ti,ab,kw OR Microbiota:ti,ab,kw OR mycobiome:ti,ab,kw

OR

'probiotic agent'/exp OR 'synbiotic agent'/exp OR 'intestine flora'/exp OR 'mycobiome'/exp)

AND

('psychiatr*':ti,ab,kw OR 'mental disorder':ti,ab,kw OR 'mental health':ti,ab,kw OR 'depress*':ti,ab,kw OR 'psychot*':ti,ab,kw

OR

'psychiatry'/exp OR 'mental disease'/exp OR 'mental health'/exp)

**SCOPUS**

("side effect" OR "adverse event" OR "metabolic syndrome" OR metabolism OR "weight change" OR "weight gain" OR "weight loss" OR diabetes OR cholesterol OR hyperlipidemia OR dyslipidemia OR hba1c OR overnutr* OR malnutr* OR obes*)

AND

(pharmacolog* OR pharmaceutic* OR medication OR "medication adherence" psychopharm* OR antidepress* OR neuroleptic OR antipsychotic)

AND

(probiotic OR synbiotic OR Microbiome OR Microbiota OR mycobiome)

AND

(Psychiatry OR mental health OR mental disorders OR depress* OR psychot*)

**Web of Science**

("side effect" OR "adverse event" OR "metabolic syndrome" OR metabolism OR "weight change" OR "weight gain" OR "weight loss" OR diabetes OR cholesterol OR hyperlipidemia OR dyslipidemia OR hba1c OR overnutr* OR malnutr* OR obes*)

AND

(pharmacolog* OR pharmaceutic* OR medication OR "medication adherence" psychopharm* OR antidepress* OR neuroleptic OR antipsychotic)

AND

(probiotic OR synbiotic OR Microbiome OR Microbiota OR mycobiome)

AND

(Psychiatr* OR mental health OR mental disorders OR depress* OR psychot*)

**Pubmed**

("side effect"[Title/Abstract] OR "adverse event"[Title/Abstract] OR "metabolic syndrome"[Title/Abstract] OR "metabolism"[Title/Abstract] OR "weight change"[Title/Abstract] OR "weight gain"[Title/Abstract] OR "weight loss"[Title/Abstract] OR "Diabetes"[Title/Abstract] OR "cholesterol"[Title/Abstract] OR "Hyperlipidemia"[Title/Abstract] OR "Dyslipidemia"[Title/Abstract] OR "hba1c"[Title/Abstract] OR "overnutr*"[Title/Abstract] OR "malnutr*"[Title/Abstract] OR "obes*"[Title/Abstract]

OR

"drug related side effects and adverse reactions"[MeSH Terms] OR "metabolic side effects of drugs and substances"[MeSH Terms] OR "metabolic syndrome"[MeSH Terms] OR "metabolism"[MeSH Terms] OR "glucose metabolism disorders"[MeSH Terms] OR "lipid metabolism disorders"[MeSH Terms] OR "weight gain"[MeSH Terms] OR "weight loss"[MeSH Terms] OR "body weight changes"[MeSH Terms] OR "diabetes mellitus"[MeSH Terms] OR "hyperlipidemias"[MeSH Terms] OR "dyslipidemias"[MeSH Terms] OR "glycated hemoglobin a"[MeSH Terms] OR "Overnutrition"[MeSH Terms] OR "Malnutrition"[MeSH Terms])

AND

("pharmacolog*"[Title/Abstract] OR "pharmaceutic*"[Title/Abstract] OR "Medication"[Title/Abstract] OR "medication adherence"[Title/Abstract] OR "psychopharm*"[Title/Abstract] OR "antidepress*"[Title/Abstract] OR "neuroleptic"[Title/Abstract] OR "antipsychotic"[Title/Abstract]

OR

"pharmacology"[MeSH Terms] OR "pharmaceutical preparations"[MeSH Terms] OR "Medication Adherence"[MeSH Terms] OR "drug therapy"[MeSH Terms] OR "psychopharmacology"[MeSH Terms] OR "antidepressive agents"[MeSH Terms] OR "antipsychotic agents"[MeSH Terms])

AND

("probiotic*"[Title/Abstract] OR "synbiotic*"[Title/Abstract] OR "Microbiome"[Title/Abstract] OR "Microbiota"[Title/Abstract] OR "Mycobiome"[Title/Abstract]

OR

"probiotics"[MeSH Terms] OR "synbiotics"[MeSH Terms] OR "gastrointestinal microbiome"[MeSH Terms] OR "microbiota"[MeSH Terms] OR "mycobiome"[MeSH Terms])

AND

("psychiatr*"[Title/Abstract] OR "mental health"[Title/Abstract] OR "mental disorders"[Title/Abstract] OR "depress*"[Title/Abstract] OR "psychot*"[Title/Abstract]

OR

"psychiatry"[MeSH Terms] OR "mental disorders"[MeSH Terms] OR "mental health"[MeSH Terms])
